# Next-generation dendritic cells for immunotherapy of acute myeloid leukemia

**DOI:** 10.1186/2051-1426-2-S3-P84

**Published:** 2014-11-06

**Authors:** Frauke Schnorfeil, Felix Lichtenegger, Christiane Geiger, Thomas Köhnke, Veit Bücklein, Torben Altmann, Beate Wagner, Reinhard Henschler, Iris Bigalke, Gunnar Kvalheim, Wolfgang Hiddemann, Dolores Schendel, Marion Subklewe

**Affiliations:** 1 Clinical Cooperation Group Immunotherapy, Helmholtz Zentrum München, Germany; 2 Department of Internal Medicine III, Klinikum der Universität München, Germany; 3 Institute of Molecular Immunology, Helmholtz Zentrum München, Germany; 4 Department of Internal Medicine III, Klinikum der Universität München, and Clinical Cooperation Group Immunotherapy at the Helmholtz Institute Munich, Germany; 5 Department of Transfusion Medicine, Cellular Therapeutics and Hemostaseology, Klinikum der Universität München, Germany; 6 Department of Cellular Therapy, The Norwegian Radium Hospital, Oslo University Hospital, Norway

## 

Post-remission therapy of patients with acute myeloid leukemia (AML) is critical for the elimination of minimal residual disease (MRD) and a prerequisite for achieving cure. Cellular immunotherapy is a highly effective treatment option as demonstrated by the low relapse rate after allogeneic stem cell transplantation (SCT). However, many patients are not eligible for this treatment. Therapeutic vaccination with autologous dendritic cells (DCs) is a promising strategy to induce anti-cancer immune responses. We have developed a GMP-compliant protocol for the generation of *next-generation *DCs. A short 3-day differentiation period is combined with a novel maturation cocktail including a TLR7/8 agonist, resulting in DCs characterized by a positive co-stimulatory profile, high production of IL-12p70, polarization of T helper cells into Th1 and efficient stimulation of cytotoxic T lymphocytes and NK cells.

In a current proof-of-concept Phase I/II clinical trial we evaluate *next-generation *DCs as post-remission therapy for AML patients with a non-favorable risk profile (NCT01734304). Standard exclusion criteria apply, and patients have to be ineligible for allogeneic SCT. DCs are generated from patients´ monocytes and loaded with RNA encoding the leukemia-associated antigens WT1, PRAME or CMVpp65 as an adjuvant and surrogate antigen. Patients are vaccinated intradermally with 5x10^6 ^DCs of each of the three different batches up to 10 times within 26 weeks. Primary endpoints are feasibility and safety, and secondary endpoints include immune responses and disease control with a particular focus on MRD conversion. Phase I will include 6 patients and Phase II another 14 patients.

So far, three patients have been enrolled and two of them have been vaccinated for at least six times each. DCs fulfilled all quality criteria (phenotype, viability, sterility, cell count, purity), and after thawing maintained their positive co-stimulatory profile as well as their capacity to secrete high amounts of IL-12p70. DCs expressed all three antigens and were able to induce a selective T cell response *in vitro*, suggesting proper antigen processing and presentation. In both vaccinated patients delayed type hypersensitivity reactions developed. Apart from erythema and itching at the injection site, no higher grade adverse events have been observed. As of yet, both patients are relapse-free and MRD-negative. Up-to-date clinical and immunomonitoring data including evaluation of T and NK cell activation and specific T cell responses will be presented.

